# Physiological and Metabolic Effects of the Inoculation of Arbuscular Mycorrhizal Fungi in *Solanum tuberosum* Crops under Water Stress

**DOI:** 10.3390/plants11192539

**Published:** 2022-09-27

**Authors:** Analía Valdebenito, Javiera Nahuelcura, Christian Santander, Pablo Cornejo, Boris Contreras, Sergio Gómez-Alonso, Antonieta Ruiz

**Affiliations:** 1 Departamento de Ciencias Químicas y Recursos Naturales, Scientific and Technological Bioresource Nucleus BIOREN-UFRO, Universidad de La Frontera, Avda. Francisco Salazar 01145, Temuco 4811230, Chile; 2 Escuela de Agronomía, Facultad de Ciencias Agronómicas y de los Alimentos, Pontificia Universidad Católica de Valparaíso, Quillota 2260000, Chile; 3 Novaseed Ltda. and Papas Arcoiris Ltda., Loteo Pozo de Ripio s/n, Parque Ivian II, Puerto Varas 5550000, Chile; 4 Instituto Regional de Investigación Científica Aplicada, Universidad de Castilla-La Mancha, 13001 Ciudad Real, Spain

**Keywords:** antioxidant activity, arbuscular mycorrhizal fungi, potato, usable humidity, water stress

## Abstract

*Solanum tuberosum* is one of the most important crops in the world; however, drought has caused significant losses in its production. One solution is the use of arbuscular mycorrhizal fungi (AMF). In this study, the phenolic profiles and antioxidant activity of the leaves of two potato genotypes (VR808 and CB2011-104) were evaluated over time in crops inoculated with two strains of AMF, as well as a consortium, in combination with a commercial fungicide. In addition, three usable humidity levels were established after the beginning of tuberization. The phenolic compounds found during the first sampling time in the VR808 genotype reached a maximum of 3348 mg kg^−1^, and in the CB2011-104 genotype, they reached a maximum of 2982 mg kg^−1^. Seven phenolic compounds were detected in the VR808 genotype, and eleven were detected in the CB2011-104 genotype, reaching the highest concentration at the last sampling time. The antioxidant activity in the first sampling was greater than the Trolox equivalent antioxidant capacity (TEAC), and in the third sampling, the cupric reducing antioxidant capacity (CUPRAC) predominated. The association of AMF with the plant by PCA demonstrated that these fungi assist in protecting the plants against water stress, since in the last harvest, the results were favorable with both mycorrhizae.

## 1. Introduction

The potato *(Solanum tuberosum*) is one of the most widely grown crops around the world, and it has a beneficial nutritional impact [1], as the fourth most frequently grown crop in terms of production after rice, wheat, and corn [2]. Currently, it is cultivated in 149 countries [3] and is important for food security, thus various studies have been performed to evaluate its response under extreme conditions [4]. Global warming has significantly affected crop growth and yield, and therefore poses a great threat to agricultural food production [5]. The potato is grown primarily in relatively cold climates and is sensitive to drought due to its shallow root system [1]; it has an optimal growth temperature of 20–25 °C, and it requires a temperature of approximately 15–25 °C for tuberization [6].

Plants are constantly exposed to extreme conditions that cause stress, which affects their growth, development, and production. These conditions include abiotic factors, such as deficiency, or overabundance conditions such as drought, temperature, salinity, light, nutrients, flooding, and phytotoxic compounds [4]. Stress activates a wide range of plant responses, such as changes in gene expression and cell metabolism, which affect growth rate and crop yield [7]. Compared to other crops, potatoes are more sensitive to drought stress [8]. Drought can cause yield losses of 13% to 94% depending on intensity and duration [9], when water deficiency occurring specifically during the tuber growth period reduces the yield to a higher extent than water limitation during other stages of potato growth [2]. The measurement of lipid peroxidation through the formation of malondialdehyde (MDA) can be a way to evaluate the oxidative stress [10] caused by drought.

Arbuscular mycorrhizal fungi (AMF) are soil microorganisms that have existed for more than 400 million years and are morphologically unchanged [11]. Mycorrhizal symbiosis is a mutually beneficial association between plant roots and soil fungi. AMF initiate a symbiotic union with the roots of 80% of terrestrial crops [12]. AMF intimately connect plants to the fungal hyphal network [11] and improve the absorption of nutrients such as phosphorus and water [13]. In exchange for supplying these components to plants, AMF obtain carbohydrates from them [11]. Many ecophysiological studies indicate that AMF symbiosis is an essential component in helping plants cope with water demands [14]. AMF improve the water absorption of roots; they also allow the plants to maintain higher hydration. In addition, these fungi help the plants maintain the turgor of their organs, which allows for the natural activity of the cells in general to be maintained, as they are primarily linked to the photosynthetic machinery [9]. In maize plants inoculated with *Rhizophagus irregularis*, two PIP aquaporin genes (ZmPIP2;2 y ZmPIP2;6) were upregulated under drought stress, demonstrating a better performance of AMF root cells in water transport under water deficit, which is connected to the shoot physiological performance in terms of photosynthetic capacity [15]. Chitarra et al. (2016) [16] have also reported that AMF symbiosis increased stomatal conductance, net photosynthesis, and related parameters, showing a higher photosynthetic capacity in these plants. A meta-analysis carried out by Chandrasekaran et al. [17] revealed that plants inoculated with AMF under hydric stress conditions have a significant increase in growth, being up to 49% higher; they also have an increase in the absorption of nutrients, which produces better crop yield. In plants of *Robinia pseudoacacia* L., the height increased by 100% with the inoculation of AMF with respect to the non-inoculated ones [18], and the growth of the seedlings of *Pistacia vera* L. treated with AMF was greater than that of the treatment without AMF. The same was true for the contents of N and Ca [19].

Plants synthesize primary metabolites for essential functions, such as growth and development, and secondary metabolites for more specific functions under drought conditions and before other types of environmental stresses, in which secondary metabolites play an important role in the adaptation and survival of stressed plants [20]. There are several types of secondary metabolites in plants that protect and regulate functions. According to their biosynthetic pathways and chemical natures, they are classified into three main groups: phenolic compounds, terpenes, and nitrogenous compounds such as alkaloids [21]. More than thirty different types of phenolic compounds have been identified in potato leaves [22], while in the tuber, the content of these compounds depends on the variety, and they can accumulate due to environmental stresses, such as high temperatures or drought [23]. In previous studies, the presence of quercetin derivatives and chlorogenic acids has been reported in leaves [24]. In the roots, their presence is primarily due to exudates of oxalic acid, while in tubers, the contents of these compounds depend on the plant variety, highlighting the presence of chlorogenic acids, such as 5-caffeoylquinic acid, and anthocyanins, such as those derived from petunidins, [25]. Under drought stress, the total phenol content in *Solanum lycopersicum* plants increases between 50% and 83% compared to the control [26]. In wheat plants, a tendency to increase in total phenol concentrations under water stress and inoculation with AMF has been reported, where caffeoylhexoside and apigenin-C-pentoside-C-hexoside III concentrations increase 4.7% and 30.2%, respectively, as compared to the treatment without stress [27]. On the other hand, increases of up to 100% in phenolic content under drought stress conditions in *Eriocephalus africanus* plants have been reported [28]. The increase in these concentrations is because the phenolic compounds protect cells from oxidative damage [29]. Phenols are nonenzymatic antioxidants that eliminate or inhibit the oxidative action of ROS to prevent cell damage; therefore, a balance between ROS generation and antioxidant activities is important in crops under water stress [30]. In the literature, in buckwheat crops inoculated with a mix composed by *Rhizophagus fasciculatus*, *Funneliformis mosseae*, and *R. irregularis* and exposed to water stress, their concentrations of phenols and flavonoids increased, which is related to greater antioxidant properties. This increase showed that mycorrhizae act as moderators and cause the production of secondary metabolites [31]. Similarly, *Triticum aestivum* was inoculated with two AMF strains, also increasing the total phenol content by 55% and 40%, compared to the control [32]. Potato plants inoculated with AMF displayed higher growth, a higher root-to-shoot ratio, and better efficiency in the use of phosphorus after inoculation with AMF (*G. intraradices*) compared to noninoculated plants [33]. According to these antecedents, our hypothesis is that *Solanum tuberosum* plants exposed to water stress have improved physiological and metabolic activities when inoculated with AMF, and the main aim of this study is to evaluate the effect of AMF inoculation on *Solanum tuberosum* crops exposed to water stress by evaluating the physiological and metabolic parameters of the inoculated and noninoculated plants.

## 2. Results

### 2.1. Profiles and Concentrations of Phenolic Compounds

Different profiles of flavonols and HCADs were detected in the different genotypes, and three harvests were performed during growth; however, in both cases, the HCADs corresponded primarily to caffeoylquinic acid isomers and the flavonols corresponded to quercetin and kaempferol derivatives (Table S1). Quantitative determinations were performed using HPLC-DAD after the corresponding analytical validation in each case (Table 1).

**Figure 1:**
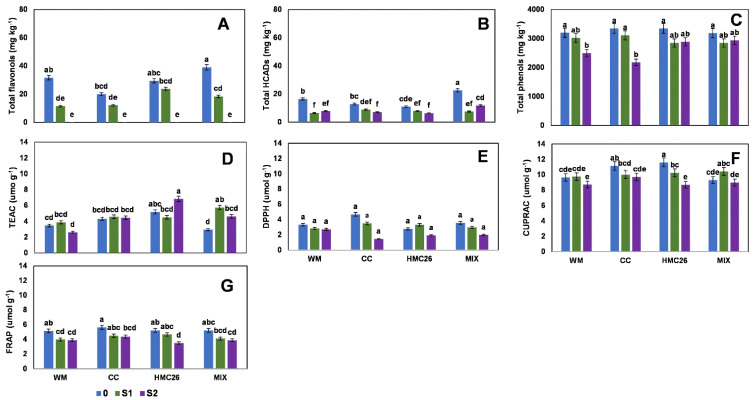
Phenolic compound concentrations and antioxidant activities of leaves of *Solanum tuberosum*, genotype VR808, under water stress and inoculation of arbuscular mycorrhizal fungi, in the first harvest. (**A**) Total flavonols by HPLC-DAD, (**B**) Total hydroxycinnamic acids by HPLC-DAD, (**C**) Total phenols determined by the Folin-Ciocalteu method, (**D**) Antioxidant activity (AA) determined by the TEAC (Trolox equivalent antioxidant capacity) method, (**E**) AA determined by the DPPH (2,2-diphenyl-1-picrylhydrazyl) method, (**F**) AA determined by the CUPRAC (copper reducing antioxidant capacity) method and (**G**) AA determined by the FRAP (ferric reducing antioxidant power) assay. Means followed by the same lowercase letter compare the difference in stress level within the same inoculation condition (Tukey 5%). Where, WM: without mycorrhiza inoculation, CC: *Claroideoglomus claroideum*, HMC26: *Claroideoglomus lamellosum* and MIX: CC + HMC26; 0: normal irrigation; S1 and S2: levels of water stress.

**Figure 2:**
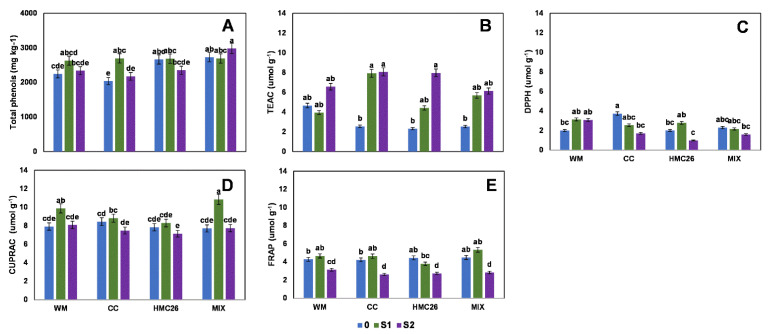
Phenolic compounds and antioxidant activity of leaves of *Solanum tuberosum*, genotype CB2011-104, under water stress and inoculation of arbuscular mycorrhizal fungi, in the first harvest. (**A**) Total phenols determined by the Folin-Ciocalteu method, (**B**) Antioxidant activity (AA) determined by the TEAC (Trolox equivalent antioxidant capacity) method, (**C**) AA determined by the DPPH (2,2-diphenyl-1-picrylhydrazyl) method, (**D**) AA determined by the CUPRAC (copper reducing antioxidant capacity) method, and (**E**) AA determined by the FRAP (ferric reducing antioxidant power) assay. Means followed by the same lowercase letter compare the difference in stress level within the same inoculation condition (Tukey 5%). Where, WM: without mycorrhiza inoculation, CC: *Claroideoglomus claroideum*, HMC26: *Claroideoglomus lamellosum* and MIX: CC + HMC26; 0: normal irrigation; S1 and S2: levels of water stress.

Regarding the first harvest, in the VR808 genotype, only one flavonol and one HCAD were detected, in which the concentrations varied from 11.4 to 39.0 mg kg^−^^1^ and 6.5 to 22.5 mg kg^−^^1^, respectively, and the maximum value in both was from the MIX treatment (Figure 1A,B, Figures S1A and S2A). However, the treatments containing mycorrhizae tended to decrease with respect to the control value in all treatments with stress. For the total phenols (Figure 1C), the highest concentration was 3348 mg kg^−^^1^ in the CC treatment, with the highest concentration observed during this study, with a tendency to decrease with or without mycorrhizae when water stress increases. In the same harvest, in the CB2011-104 genotype, individual flavonols and HCADs were undetected (Figures S3A and S4A), whereas the total phenols (Figure 2A) reached a similar concentration relative to that of the VR808 genotype, since its highest concentration was 2982 mg kg^−^^1^ in the MIX treatment.

**Figure 3:**
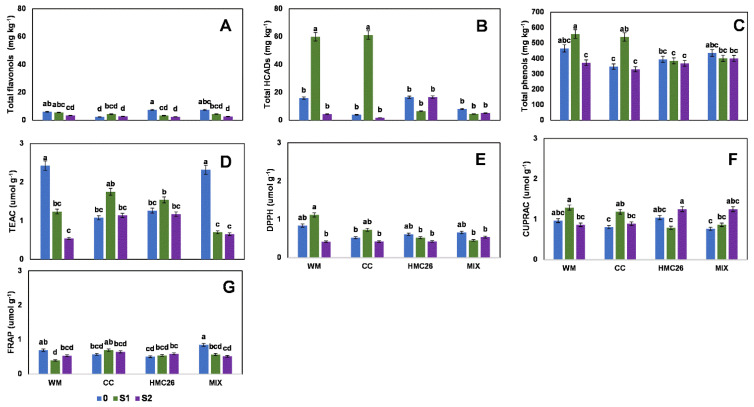
Phenolic compounds and antioxidant activities of leaves of *Solanum tuberosum*, genotype VR808, under water stress and inoculation of arbuscular mycorrhizal fungi, in the second harvest. (**A**) Total flavonols by HPLC-DAD, (**B**) Total hydroxycinnamic acids by HPLC-DAD, (**C**) Total phenols determined by the Folin-Ciocalteu method, (**D**) Antioxidant activity (AA) determined by the TEAC (Trolox equivalent antioxidant capacity) method, (**E**) AA determined by the DPPH (2,2-diphenyl-1-picrylhydrazyl) method, (**F**) AA determined by the CUPRAC (copper reducing antioxidant capacity) method and (**G**) AA determined by the FRAP (ferric reducing antioxidant power) assay. Means followed by the same lowercase letter compare the difference in stress level within the same inoculation condition (Tukey 5%). Where, WM: without mycorrhiza inoculation, CC: *Claroideoglomus claroideum*, HMC26: *Claroideoglomus lamellosum* and MIX: CC + HMC26; 0: normal irrigation; S1 and S2: levels of water stress.

**Figure 4:**
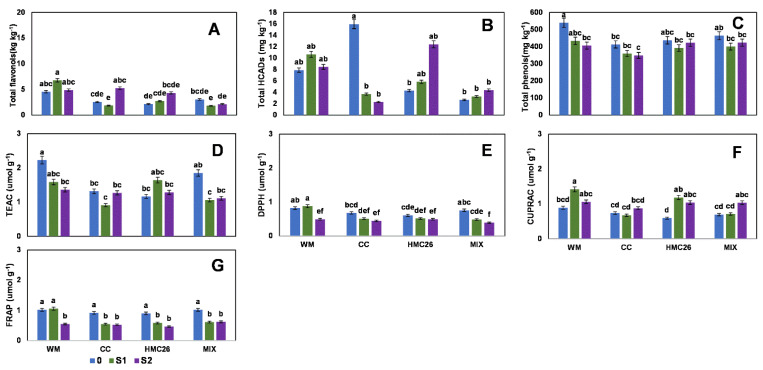
Phenolic compounds and antioxidant activities of leaves of *Solanum tuberosum*, genotype CB2011-104, under water stress and inoculation of arbuscular mycorrhizal fungi, in the second harvest. (**A**) Total flavonols by HPLC-DAD, (**B**) Total hydroxycinnamic acids by HPLC-DAD, (**C**) Total phenols determined by the Folin-Ciocalteu method, (**D**) Antioxidant activity (AA) determined by the TEAC (Trolox equivalent antioxidant capacity) method, (**E**) AA determined by the DPPH (2,2-diphenyl-1-picrylhydrazyl) method, (**F**) AA determined by the CUPRAC (copper reducing antioxidant capacity) method and (**G**) AA determined by the FRAP (ferric reducing antioxidant power) assay. Means followed by the same lowercase letter compare the difference in stress level within the same inoculation condition (Tukey 5%). Where, WM: without mycorrhiza inoculation, CC: *Claroideoglomus claroideum*, HMC26: *Claroideoglomus lamellosum* and MIX: CC + HMC26; 0: normal irrigation; S1 and S2: levels of water stress.

For the second harvest, in the VR808 genotype, one flavonol and two HCADs were detected (Figures S1B and S2B, Table S2). The concentration of total flavonols decreased considerably compared to the first harvest, with a range of between 2.4 mg kg^−^^1^ and 7.5 mg kg^−^^1^, with the highest value detected in the control treatment with HMC26 and MIX inoculation, displaying this trend when stress increases under mycorrhizae inoculation. However, the HCADs increased considerably in the uninoculated (WM) and CC treatments with S1 stress, with increases of 380% and 1560%, respectively, as compared to the control with normal irrigation (Figure 3A,B). The HCAD with the highest concentration was 5-caffeoylquinic acid, at 59.7 mg kg^−^^1^. The total phenols (Figure 3C) also decreased compared to the first harvest, presenting a range of between 347.4 and 558.2 mg kg^−^^1^, in which the highest concentration was detected in the WM and CC treatments with stress at the S1 level; however, the HMC26 and MIX treatments showed a tendency to decrease due to stress, but the changes were not significant. During the same harvest, in the CB2011-104 genotype, two flavonols and two HCADs were detected (Figures S3B and S4B, Table S3), and the flavonols (Figure 4A) showed a tendency to decrease under stress, primarily in S1, but there were no significant differences among the different treatments. However, a higher proportion of acids was detected, reaching a maximum concentration of 15.9 mg kg^−^^1^ (Figure 4B) in the treatment under CC inoculation with normal irrigation, with decreases of 77% and 85% under stresses S1 and S2, respectively. On the other hand, total phenols with treatments under inoculation with HMC26 and MIX (Figure 4C) showed a tendency to decrease with greater stress.

**Figure 5:**
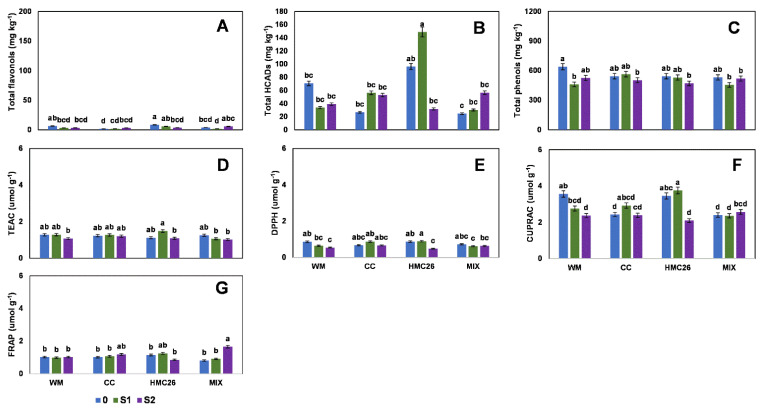
Phenolic compounds and antioxidant activities of leaves of *Solanum tuberosum*, genotype VR808, under water stress and inoculation of arbuscular mycorrhizal fungi, in the third harvest. (**A**) Total flavonols by HPLC-DAD, (**B**) Total hydroxycinnamic acids by HPLC-DAD, (**C**) Total phenols determined by the Folin-Ciocalteu method, (**D**) Antioxidant activity (AA) determined by the TEAC (Trolox equivalent antioxidant capacity) method, (**E**) AA determined by the DPPH (2,2-diphenyl-1-picrylhydrazyl) method, (**F**) AA determined by the CUPRAC (copper reducing antioxidant capacity) method and (**G**) AA determined by the FRAP (ferric reducing antioxidant power) assay. Means followed by the same lowercase letter compare the difference in stress level within the same inoculation condition (Tukey 5%). Where, WM: without mycorrhiza inoculation, CC: *Claroideoglomus claroideum*, HMC26: *Claroideoglomus lamellosum* and MIX: CC + HMC26; 0: normal irrigation; S1 and S2: levels of water stress.

**Figure 6:**
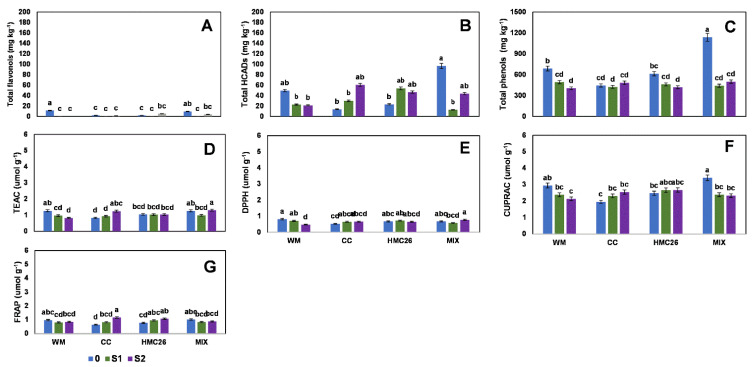
Phenolic compounds and antioxidant activities of leaves of *Solanum tuberosum*, genotype CB2011-104, under water stress and inoculation of arbuscular mycorrhizal fungi, in the third harvest. (**A**) Total flavonols by HPLC-DAD, (**B**) Total hydroxycinnamic acids by HPLC-DAD, (**C**) Total phenols determined by the Folin-Ciocalteu method, (**D**) Antioxidant activity (AA) determined by the TEAC (Trolox equivalent antioxidant capacity) method, (**E**) AA determined by the DPPH (2,2-diphenyl-1-picrylhydrazyl) method, (**F**) AA determined by the CUPRAC (copper reducing antioxidant capacity) method and (**G**) AA determined by the FRAP (ferric reducing antioxidant power) assay. Means followed by the same lowercase letter compare the difference in stress level within the same inoculation condition (Tukey 5%). Where, WM: without mycorrhiza inoculation, CC: *Claroideoglomus claroideum*, HMC26: *Claroideoglomus lamellosum* and MIX: CC + HMC26; 0: normal irrigation; S1 and S2: levels of water stress.

In the third harvest, the VR808 genotype, only one flavonol and five HCADs were detected (Figures S1C and S2C). The flavonols (Figure 5A) followed a trend of maintaining similar concentrations compared to the second harvest, and their concentrations varied between 1.6 and 8.6 mg kg^−^^1^. At this harvest, the HCADs presented a considerable increase (Figure 5B) compared to the second harvest. In the HMC26 treatment, an increase of 54% was detected in HCAD concentrations compared to the control with normal irrigation; HCAD6 (Table S4) is responsible for the increase in the S1 level with the HMC26 treatment, reaching a concentration of 148.8 mg kg^−^^1^. The total phenols (Figure 5C) maintained their range of concentrations, without considerable variations compared to the previous harvest, showing a tendency to decrease in the different treatments under stress. In the CB2011-104 genotype, four flavonols and four HCADs were detected (Figures S3C and S4C), showing the same trend as the VR808 genotype, since their total concentrations ranged from 1.4 to 11.4 mg kg^−^^1^ and 13.7 to 96.8 mg kg^−^^1^, respectively (Figure 6A,B). The acids that produced this increase were 5-caffeoylquinic acid and HCAD6 (Table S5). However, within the total phenol concentrations (Figure 6C), a decrease in the S1 level of 64% of the MIX treatment was observed compared to the other treatments showing a decrease with greater stress, but the differences were not significant.

The *p*-values demonstrating the significance of the mycorrhizal inoculant type, water stress level, and their interaction for concentrations of phenolic compounds are shown in the supplementary material tables for both *Solanum tuberosum* genotypes in the three stages of harvest (Tables S6–S11).

Antioxidant capacity is an essential index for evaluating the defense system of the plant, since it contributes to a more complete study when analyzing information related to the elimination of free radicals, for which methods such as CUPRAC (copper reducing antioxidant capacity), FRAP (ferric reducing antioxidant power) assay, TEAC (Trolox equivalent antioxidant capacity), and DPPH (2,2-diphenyl-1-picrylhydrazyl) method are widely used (Table 1).

In the first harvest, the antioxidant activity of the VR808 genotype (Figure 1D–F) showed a tendency to decrease as stress increased; however, TEAC and CUPRAC showed higher proportions, reaching concentrations between 2.6 and 6.8 µmol g^−^^1^ and 8.7 and 11.6 µmol g^−^^1^, respectively, in which the highest values were detected in the CC and HMC26 treatments; however, in the TEAC, there were no significant differences between the treatments. In the CB2011-104 genotype (Figure 2B–E), TEAC and CUPRAC also showed higher concentrations, but in TEAC, a tendency to increase under stress was observed, highlighting the CC and HMC26 treatments, in which there were increases of 320% and 340% at the S2 level compared to the controls with normal irrigation, whereas in the other treatments, a tendency to decrease at higher stress was observed.

During the second harvest, the antioxidant activity levels in the VR808 genotype (Figure 3D–G) in TEAC showed a decrease under stress, indicating a decrease of 71.7% in the MIX treatment with the higher stress level. In addition, under the other methods, the antioxidant activity showed the same tendency to decrease under stress. In the CB2011-104 genotype (Figure 4D–G), the highest levels were detected under the TEAC method (0.9 to 2.2 µmol g^−^^1^). In addition, in all the antioxidant activity methods, a tendency to decrease with S1 and S2 stresses was observed; however, primarily in S1, there were no significant differences compared to the control with normal irrigation.

In contrast, in the third harvest, the CUPRAC values increased again in both genotypes. In the VR808 genotype (Figure 5D–G), the CUPRAC levels reached a maximum of 3.7 µmol g^−^^1^ in the HMC26 treatment with a stress level of S1, but there were no significant differences between the treatments for this increase. TEAC, DPPH, and FRAP maintained their concentrations in ranges similar to the second harvest, showing a tendency to decrease with increasing stress. In genotype CB2011-104 (Figure 6D,E), CUPRAC had concentrations between 1.9 and 3.4 µmol g^−^^1^, with the highest value detected in the MIX treatment. The other methods remained at similar levels compared to the VR808 genotype, since no significant differences were detected between the treatments at the three test levels, but rather they were maintained at the same concentrations.

Regarding lipid peroxidation (Figure S5), in the third harvest, for the VR808 genotype with HMC26 treatment, a tendency to decrease could be observed under both stress conditions, in addition to showing a profile similar to that obtained in DPPH. For the CB2011-104 genotype, this trend could be observed in the WM and MIX treatments and was higher in the latter. Lastly, in both the second and third harvests, a similar profile was observed in the CB2011-104 genotype with the HMC26 treatment between the concentrations of MDA and CUPRAC.

The *p*-values signifying the mycorrhizal inoculant type, water stress level, and their interaction for concentrations of the antioxidant activity are showed in supplementary material tables for both *Solanum tuberosum* genotypes in the three stages of harvest (Tables S6–S13).

### 2.2. Overall Results

**Figure 7:**
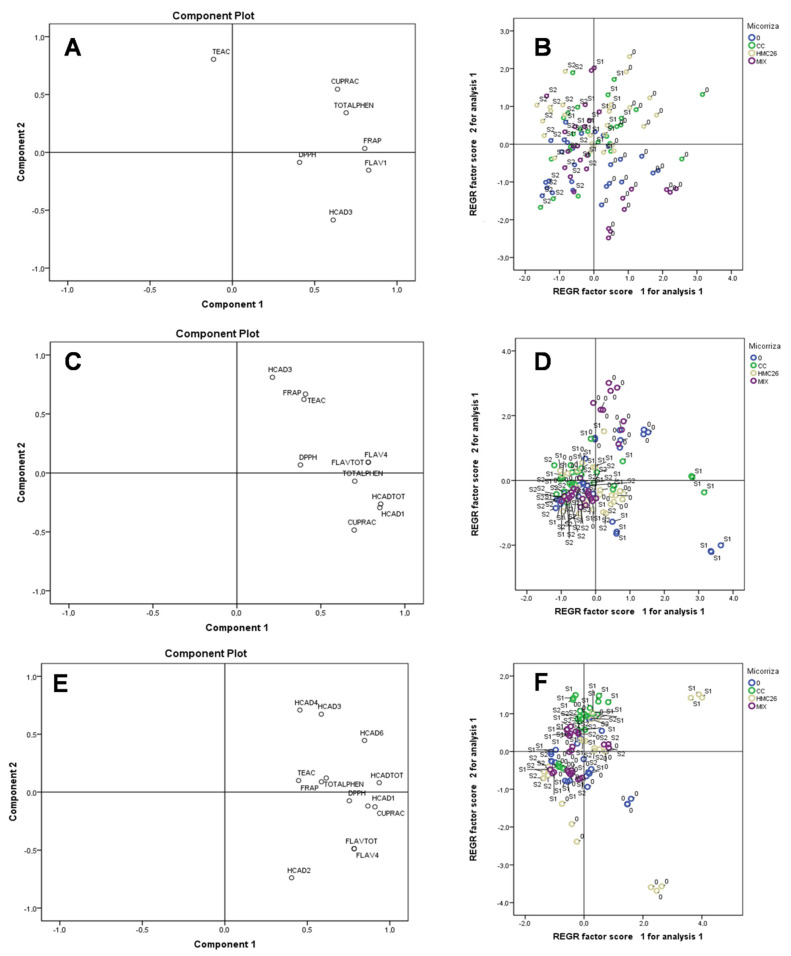
Principal component (PC) scores for the experimental variables (left) determined in *Solanum tuberosum* leaves, and the grouping of the samples according to the distribution of PCs (right) of the VR808 genotype. First harvest (**A**,**B**), second harvest (**C**,**D**), third harvest (**E**,**F**). Where: HCAD1, HCAD2, HCAD3: individual hydroxycinnamic acids; FLAV1, FLAV2, FLAV3, FLAV4: individual flavonols; FLAVTOT: total flavanols; HCADTOT: total hydroxycinnamic acids; TOTALPHEN: concentration of total phenolics according to Folin–Ciocalteu method; TEAC: Trolox equivalent antioxidant capacity; CUPRAC: reducing antioxidant capacity of the cupric ion; DPPH: antioxidant activity of the DPPH radical; FRAP:, ferric reducing antioxidant power; 0: without arbuscular mycorrhizal fungi; CC: *Claroideoglomus claroideum*; HMC26: *Claroideoglomus lamellosum*; MIX *Claroideoglomus claroideum + Claroideoglomus lamellosum*; S1 and S2: levels of water stress.

A principal component analysis (PCA) was performed considering all the evaluated parameters. In the VR808 genotype of the first harvest (Figure 7A,B), the most representative variables for PC1 were total phenols, CUPRAC, and FRAP. In addition, a high correlation was observed at the level without stress (0) with the CC mycorrhiza and HMC26. This finding may indicate that the interaction of AMF with the plant is beneficial for the antioxidant response and defense system in this genotype. In the second harvest (Figure 7C,D), the most representative variables were FRAP, TEAC, DPPH, HCAD3, and total flavonols, in which the treatments without stress and without AMF were grouped positively, but those inoculated with the mixture of mycorrhizae (MIX) were also important due their relation between phenolic concentrations and antioxidant activity. In the third harvest (Figure 7E,F), the most representative variables of PC1 were HCADs, total phenols, TEAC, and FRAP, as explained by treatments with a stress level of S1, together with CC and HMC26 mycorrhizae inoculation.

**Figure 8:**
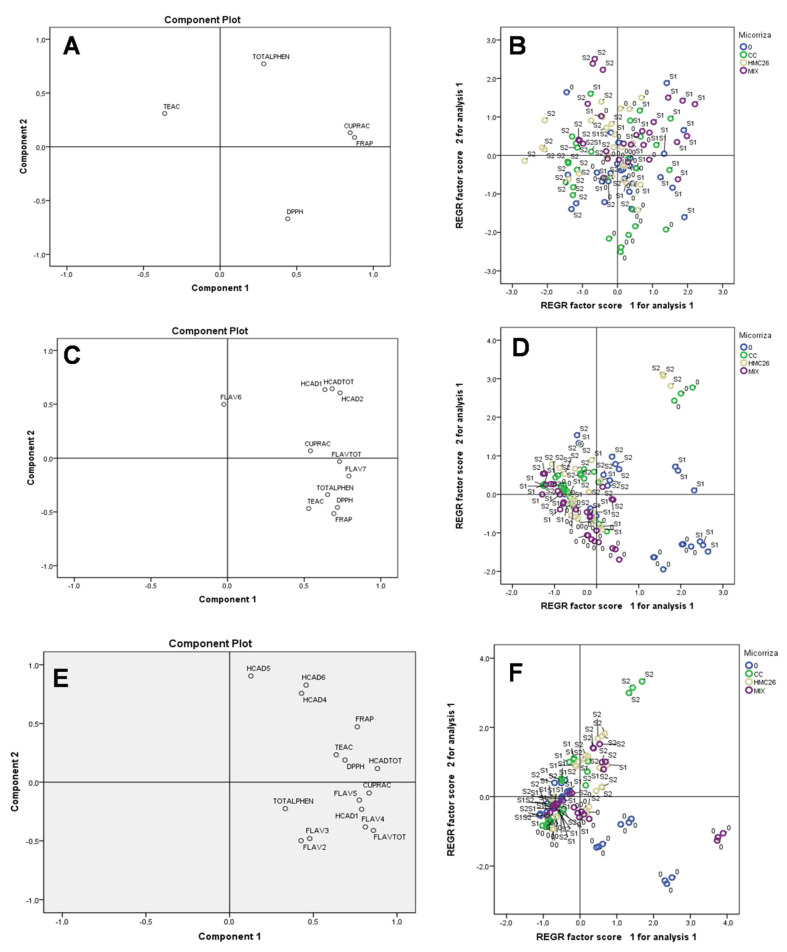
Principal component (PC) scores for the experimental variables (left) determined in *Solanum tuberosum* leaves and the grouping of the samples according to the distribution of PCs (right), of the genotype CB2011-104. First harvest (**A**,**B**), second harvest (**C**,**D**), third harvest (**E**,**F**). Where: HCAD1, HCAD2, HCAD3: individual hydroxycinnamic acids; FLAV1, FLAV2, FLAV3, FLAV4: individual flavonols; FLAVTOT: total flavanols; HCADTOT: total hydroxycinnamic acids; TOTALPHEN: concentration of total phenolics according to Folin–Ciocalteu method; TEAC: Trolox equivalent antioxidant capacity; CUPRAC: reducing antioxidant capacity of the cupric ion; DPPH: antioxidant activity of the DPPH radical; FRAP:, ferric reducing antioxidant power; 0: without arbuscular mycorrhizal fungi; CC: *Claroideoglomus claroideum*; HMC26: *Claroideoglomus lamellosum*; MIX *Claroideoglomus claroideum + Claroideoglomus lamellosum*; S1 and S2: levels of water stress.

However, during the first harvest of the CB2011-104 genotype (Figure 8A,B), the most representative variables of PC1 were total phenols, CUPRAC, and FRAP, in which the stress level S1 was predominant under inoculation with CC and MIX strains. This mycorrhiza increases the content of phenolic compounds that are responsible for the antioxidant activity by CUPRAC and FRAP. In the second harvest (Figure 8C,D), there was no clear trend, but the compounds that contributed the most were the HCADs, and they contributed to the antioxidant activity by the CUPRAC method. However, in the third harvest (Figure 8E,F), a similar trend to the second harvest, in which HCADs had a clear presence, was observed, in which the stress level S2 under inoculation with CC, HMC26, and MIX mycorrhizae protected the CB2011-104 genotype with respect to the HCAD content and antioxidant activity in the leaves. However, in the treatments without stress (Figure 8F), this effect is correlated with individual and total flavonol concentrations.

## 3. Discussion

Previous studies in other substrates, such as peat, reported higher significant differences between the various evaluated parameters. In addition, different profiles and higher concentrations of phenolic compounds have been detected in potatoes depending on the genotype, AMF, or fungicide [24]. Subsequently, the substrate produces an important difference in the expression of flavonols and HCADs. On the other hand, different trends have been reported, depending on the potato genotype and the evaluated vegetal material using commercial fungicides. For example, in tubers, the existence of functional compatibility was observed, where the optimal combinations for antioxidant response, mycorrhization degree, and performance were *C. lamellosum*/REFLECTXTRA^®^ for VR808 genotype, *F. mosseae*/MONCUT for CB2011-509 genotype, and *C. lamellosum*/MONCUT for CB2011-104 genotype [25]. On the other hand, in leaves, *C. lamellosum* and REFLECTXTRA^®^ was the ideal combination in all genotypes [24]. Notably, similar concentrations of total phenols in our first harvest of leaves in both genotypes have been reported, but the difference in this study was that only one measurement was performed beginning at the senescence stage [24]. The decrease in total phenols, up to 80% in subsequent harvests, is comparable with other crops, such as *Solanum lycopersicum* with differentiated irrigation, in which the concentrations of total phenols over time also showed a significant decrease of 83% [34]. This decrease could be caused by a redistribution of the compounds, since in the first harvest, there is a strong defense system in the leaves, which decreases after tuberization due to the filling of the tubers, which has been observed in various *Solanum tuberosum* genotypes [35]. It is important to note that in potato tubers, high concentrations of phenolic compounds, which range between 2510 and 4486 mg kg^−^^1^, and between 3891 and 3753 mg kg^−^^1^ in leaves, have been reported, which is related to our results, since in the first harvest of the VR808 genotype, a maximum of 3348 mg kg^−^^1^ was obtained, and in the CB2011-104 genotype, a maximum of 2982 mg kg^−^^1^ was obtained [24,25]. Interestingly, the variation in the phenolic content of *Scabiosa maritima* during development can be explained by the biochemical changes that occur during growth, which are related to the plant’s defenses against biotic and abiotic stresses [36]. Water deficit negatively affects the vegetative development of the plant, especially the development of the aerial part, which limits photosynthesis and provides the resources to produce tubers [37]. All stages of potato growth are sensitive to irrigation deficit; however, the most affected stage is tuber initiation, in which the early vegetative and maturity stages are considered tolerant to drought stress [38]. The plants inoculated with AMF, primarily *C. claroideum*, showed a tendency to increase the concentrations of phenolic compounds, which is one of the important parameters in the tuber stage, regardless of drought stress during the first harvest. These levels were similar. The reason why these levels were lower in the following harvests is because this change occurred when the tuberization process began, which is explained by the high concentration of these compounds in the tuber analysis [24,25]. Phenols act as a defense mechanism against herbivores, microorganisms, and competing plants [39]. In wheat plants, under AMF inoculation, the concentrations of total phenols depend on the genotype, physiological, and environmental factors, as well as the phenological stage of the crop [27,40,41]. The accumulation of phenols is very important to counteracting the negative impacts generated by drought stress in plants [42]. The main reason for this accumulation of phenolic compounds induced by drought is the modulation of the biosynthetic pathway of phenylpropanoids, as drought regulates many key genes encoding the main enzymes of the phenylpropanoid pathway, resulting in stimulated synthesis of phenolic compounds [43].

The antioxidant activity results are consistent with those reported by Fritz et al. [24], in which the activities of the TEAC, FRAP, DPPH, and CUPRAC methods were determined in potato plants inoculated with *C. claroideum* and HMC26, and similar antioxidant activity levels were observed in the first harvest [24]. Notably, the CUPRAC and TEAC activities in *F. ananassa* crops inoculated with *C. claroideum* also increased [44]. Most AMF species can associate with many plant species, and a single plant can be colonized by several AMF species [45]. However, plant responses can be different, which was observed in the mixture of mycorrhizae, since there are different degrees of functional compatibility between specific strains of AMF and plant species. However, in terms of antioxidant activity, this mixture did not present significant values [46]. Potato plants contain several active ingredients, and due to their specific structures, the antioxidant effect in specific antioxidant systems is also displayed differently. Therefore, using multiple measurements of antioxidant capacity allows us to observe responses to the different compounds [47]. In *Sesamum indicum* L., the effects of AMF inoculation (*F. mosseae* with *R. irregularis*) on the biomass and grain yield improved under severe water stress and were more effective than optimal irrigation conditions. This finding revealed that inoculating sesame plants with mycorrhizal fungi improved the state of water retention, and the plant could modulate and neutralize severe water stress; in addition, fewer antioxidant enzymes are required to eliminate radicals and active oxygen [48]. In *Zea mays* L. under AMF inoculation, there is a lower concentration of MDA than in uninoculated plants [49]. Another study with *T. aestivum* L. inoculated with *F. mosseae* under water stress produced improvements in the composition of phenols and antioxidant activities in the grains of the wheat plants, with an increase of up to 30.2% in the concentration of apigenin-C-pentoside-C-hexoside III under water stress with respect to normal irrigation [27]. Antioxidant activity is related to the purpose of protecting plants against the effects of drought stress, which generates a regulation of intracellular concentrations of ROS [50], the most common ROS are singlet oxygen, superoxide radicle, hydrogen peroxide, and hydroxyl radicle [43].

The CUPRAC method detects the antioxidant capacities of phenolic acids, flavonoids, carotenoids, and other polyphenols [51]. Potato plants, inoculated with AMF, showed a tendency to increase their antioxidant activities with the CUPRAC method [24]. This results also agree with the results reported in the study of *F. ananassa* inoculated with *C. claroideum*, where the antioxidant activity by this method also had an increase [44].

Highlighted here are the various most relevant results obtained, which were based on the monitoring of phenolic and antioxidant responses in the leaves of *Solanum tuberosum* plants over time. In both potato genotypes, under different levels of usable moisture, important variations in the leaves in the amounts of compounds and antioxidant activities were observed, since the VR808 genotype initially had greater activity when unstressed conditions were observed, but ultimately, its higher expression was at the S1 stress level under inoculation with each mycorrhiza separately. In the CB2011-104 genotype, in the first harvest, the treatment with S1 is highlighted. However, in the third harvest, all the mycorrhizal strains helped to maintain their antioxidant activities at the S2 stress level. The results reveal that the AMF helps protect the plant against hydric stress, since the PCA results show related symptoms; however, they help protect the CB2011-104 genotype to a greater extent because it was the one that possessed a greater protection under the S2 stress level in the last harvest.

## 4. Materials and Methods

### 4.1. Reagents

Water, methanol, acetonitrile (HPLC grade), Folin-Ciocalteu reagent, ethanol, and formic acid were obtained from Merck (Darmstadt, Germany). Phosphoric acid, TROLOX (6-hydroxy-2,5,7,8 acid tetramethylchroman-2-carboxylic acid), ABTS (2,2′-azino-bis (3-ethylbenzothiazoline-6-sulfonic acid)), DPPH (2,2-diphenyl-1-picrylhydrazyl), neocuproin (≥98%), quercetin (>95%), copper (II and III) chloride (grade pa), potassium persulfate, chlorogenic acid (≥95%), iron (III) chloride, calcium chloride, ammonium acetate, and methanol were obtained from Sigma-Aldrich (Steinheim, Germany). Sodium carbonate and TPTZ (2,4,6-tripyridyl-1,3,5-triazine) were obtained from Supelco (Darmstadt, Germany).

### 4.2. Samples

*Solanum tuberosum* plants were grown in pots subjected to water stress conditions in the greenhouse of the Department of Chemical Sciences and Natural Resources of the Universidad de la Frontera, Temuco, where they were kept under 50% artificial shade by placing a mesh over the greenhouse, with a light/dark photoperiod of 16/8 h and day/night temperatures of 25/18 °C. Tubers of two genotypes were used (VR808: white potato and CB2011-104: purple potato), with different skin and pulp colors, which were provided by the company Papas Arcoiris Ltd.a. (Puerto Varas, Chile). At sowing time, the potato tubers were inoculated with two strains of AMF according to each treatment (CC: *Claroideoglomus claroideum*; HMC26: *Claroideoglomus lamellosum*; MIX: CC + HMC26); moreover, control treatments without mycorrhizal (WM) were included. Plants inoculated with one inoculum also received the filtrate of the other inoculum. The inoculation method was carried out according to the one described previously by Fritz et al. (2022) [24]. The AM fungal inoculum was placed in the growth substrate under the tuber in amounts of 5 g per pot (approximately 700 spores per gram). Noninoculated plants received the same amount of autoclaved mycorrhizal inoculum together with a 10 mL aliquot of a filtrate (in Whatman N◦1 paper) of both AM inocula to supply a general microbial population that was free of AM fungal propagules. In addition, the commercial fungicide REFLECTXTRA^®^, as an agronomic management, was added in all treatments. The stress factor was applied under three conditions: 40% (S2), 70% (S1), and 100% (0) available soil moisture levels. After sowing, all plants were treated with normal watering until the beginning of tuberization. Watering was managed considering three usable humidities (40, 70, and 100%); moreover, the first collection time was carried out. Soil and sand were used as substrates in a 2:1 ratio, and this substrate underwent a sterilization process and was autoclaved for 20 min at 121 °C and 1 atm pressure. Each treatment was applied in triplicate (n = 3; N = 74). The potato leaf genotypes were harvested for a period of three months, during which the first harvest was 60 days after sowing (DAS), the second was 90 DAS, and finally the third was 120 DAS, and the leaves were immediately stored at −80 °C until analysis.

### 4.3. Identification and Quantification of Phenolic Compounds in Leaves

The extraction procedure and HPLC analyses were performed according to Fritz et al., (2022) [24] using high-performance liquid chromatography with diode array detection (HPLC DAD) equipped with an LC-20AT quaternary pump, a DGU-20A5R degassing unit, a CTO-20A oven, a SIL-20a autosampler, and an array of UV visible detector diodes (SPD M20A) (Shimadzu, Tokyo, Japan). Control and data collection were performed using Lab Solutions software (Shimadzu, Duisburg, Germany). Identity assignments were performed using an HPLC-DAD system coupled to a 6545-quadrupole time-of-flight (Q-ToF) mass spectrometer (Agilent, Waldbronn, Germany). The control software used here is a Mass Hunter workstation (version B.06.11). The chromatographic separation method for determining phenolic compounds, primarily hydroxycinnamic acids and flavonols, was performed based on what was reported by Fritz et al. [24] using a Kromasil Classic-Shell C_18_ column (100 × 4.6 mm, 2.5 µm) and a Novapak Waters C_18_ guard column (22 × 3.9 mm, 4 µm) at 40 °C and a flow rate of 0.55 mL min^−^^1^. The mobile phases were 92:3:5 (v:v:v) water, acetonitrile, and formic acid (mobile phase A), and 45:50:5 (v:v:v) water, acetonitrile, and formic acid (mobile phase B), with an elution gradient from 6 to 30% B for 10 min, 30 to 50% B for 9 min, and 50 to 6% B for 2 min, followed by 10 min of stabilization. The detection wavelengths were 320 nm for hydroxycinnamic acids (HCADs) and 360 nm for flavonols, using chlorogenic acid and quercetin as standards for external calibrations, respectively.

### 4.4. Total Phenols Determination by the Folin-Ciocalteu Method

The following reagents were added to a microtube in the following order: 15 µL of standard or extract, 750 µL of deionized water, 75 µL of Folin-Ciocalteu reagent, 300 µL of 20% m/v sodium carbonate, and 360 µL of deionized water. Solutions were incubated at 20 °C for 30 min in the dark; then, 250 μL of the solution was added to a 96-well plate, and the absorbance at 750 nm was obtained [44]. Chlorogenic acid was used as a standard.

### 4.5. Determination of Antioxidant Activity

To measure the antioxidant activity, the same extract used to measure the phenolic compounds in the leaves was used. The Trolox equivalents antioxidant capacity (TEAC), copper ion reducing antioxidant activity (CUPRAC), and the antioxidant activity by DPPH were evaluated [44]. The antioxidant capacity was determined by the iron reduction potential (FRAP) method as described by Jiménez-Aspee et al., (2014) [52], with some modifications. The FRAP solution was prepared by mixing 300 mM acetate buffer, 10 mM TPTZ, and 20 mM FeCl_3_ in a 10:1:1 v:v:v ratio. Then, 285 µL of FRAP solution was transferred to a 96-well plate, which was heated to 37 °C, followed by 15 µL of sample or TROLOX standard with an incubation time of 30 min. The absorbance was measured at 593 nm. The results were expressed as TROLOX equivalents. Lipid peroxidation was determined by the formation of malondialdehyde (MDA) using the methodology described by Du and Bramlage., (1992) [53]. The absorbance measurement was performed at 440, 532, and 600 nm. The results were expressed as malondialdehyde equivalents (nmol MDA mL^−^^1^).

### 4.6. Statistical Analysis

The main effects of mycorrhizal inoculant and water stress applications and their interactions for each genotype of *Solanum tuberosum* were statistically analyzed using a factorial analysis of variance (ANOVA). The data that did not meet the statistical assumptions of normality and homoscedasticity were transformed using the ln function, but the results were presented in their original measurement scale.

Treatments with significant differences were analyzed using Tukey’s HSD as a post hoc test to compare the means between treatments. The data were also subjected to a principal component analysis (PCA) to evaluate the multivariate effect of the established treatments and the relationship between the experimental variables. For all procedures, a *p* < 0.05 was considered statistically significant. For the analyses, SPSS 22.0 software (IBM Corp., Armonk, NY, USA) was used.

## 5. Conclusions

The response of two potato genotypes under deficit irrigation that was exposed during different stages of development of the *Solanum tuberosum* plant was evaluated. The objective was to analyze the physiological and metabolic responses of two genotypes with different colorations, with the VR808 genotype displaying white skin and yellow flesh and CB2011-104 displaying purple skin and flesh. The VR808 genotype had higher protection under the S1 stress level with both mycorrhizae, CC and HMC16, but separately. However, the CB2011-104 genotype under the S2 stress level had greater protection, and all the AMF were demonstrated to protect the plant from this level of stress. The different results indicate the different combinations of AMF, potato genotype, and stress level, which affected the content of phenolic compounds and antioxidant activity in the plant. Lastly, the effectiveness of using AMF to counteract drought stress, with interesting results in both genotypes, showed that CC and HMC26 mycorrhizae can defend potato plants that have been exposed to water stress. In potato crops, AMFs promote growth and increase tolerance to abiotic stress caused by water deficit, since they improve the morphology and antioxidant activity of these plants; however, these effects are linked to the selection and optimization of the inoculum of HMA; it is for this reason that inoculation turns out to be a promising future strategy for the cultivation of *Solanum tuberosum*.

## Figures and Tables

**Table 1:** Analytical parameters for the determination of phenolic compound concentrations and antioxidant activities by HPLC-DAD and spectrophotometric methods, respectively, in *Solanum tuberosum* leaves.

Method	Standard	Equation	R^2^	DL	QL	LR
HPLC	Quercetin	y = 128319x − 22693	0.995	0.89 mg L^−1^	2.98 mg L^−1^	2.98–60 mg L^−1^
HPLC	Chlorogenic acid	y = 115567x − 7883	1.000	0.06 mg L^−1^	0.18 mg L^−1^	0.18–100 mg L^−1^
FOLIN	Gallic acid	y = 0.0009x + 0.005	0.999	7.69 mg L^−1^	25.60 mg L^−1^	25 to 500 mg L^−1^
TEAC	Trolox	y = 0.4186x + 0.0147	0.994	0.07 µmol L^−1^	0.21 µmol L^−1^	0.21 to 0.17 µmol L^−1^
DPPH	Trolox	y = 0.5739x + 0.0084	0.996	0.02 µmol L^−1^	0.07 µmol L^−1^	0.07 to 0.7 µmol L^−1^
CUPRAC	Trolox	y = 4.2617x + 0.0545	0.994	0.02 µmol L^−1^	0.07 µmol L^−1^	0.07 to 0.4 µmol L^−1^
FRAP	Trolox	y = 1.7195x + 0.1976	0.997	0.01 µmol L^−1^	0.04 µmol L^−1^	0.04 to 0.4 µmol L^−1^

DL, detection limit; QL, quantification limit; LR, linear range.

## Data Availability

The data presented in this study are available on request from the corresponding author.

## Supplementary Materials

The following supporting information can be downloaded at: https://www.mdpi.com/article/10.3390/plants11192539/s1, Table S1: Identification of phenolic compounds by HPLC-DAD-MS/MS in leaves of *Solanum tuberosum* under water stress and inoculation of arbuscular mycorrhizal fungi. Figure S1: HPLC-DAD chromatogram (360 nm) for leaves of *Solanum tuberosum*, genotype VR808 under water stress and inoculation of arbuscular mycorrhizal fungi. Where: A: first harvest; B: second harvest; C: third harvest; 1: no identified; 4: quercetin-rutinoside. Figure S2: HPLC-DAD chromatogram (320 nm) for leaves of *Solanum tuberosum*, genotype VR808, under water stress and inoculation of arbuscular mycorrhizal fungi. Where: A: first harvest; B: second harvest; C: third harvest; 1: 5-caffeoylquinic acid 1; 3: caffeoylquinic acid isomer. Figure S3: HPLC-DAD chromatogram (360 nm) for leaves of *Solanum tuberosum*, genotype CB2011-104 under water stress and inoculation of arbuscular mycorrhizal fungi. Where: A: first harvest; B: second harvest; C: third harvest; 2: quercetin-rutinoside-pentoside, 3: no identified, 4: quercetin-rutinoside, 5: kampferol-rutinoside, 6: no identified, 7: no identified. Figure S4: HPLC-DAD chromatogram (320 nm) for leaves of *Solanum tuberosum*, genotype CB2011-104, under water stress and inoculation of arbuscular mycorrhizal fungi. Where: A: first harvest; B: second harvest; C: third harvest; 1: 5-caffeoylquinic acid, 2: caffeoylquinic acid isomer, 3: caffeoylquinic acid isomer, 4: caffeoylquinic acid isomer, 5: no identified, 6: no identified. Table S2: Individual flavonols (FLAV) and hydroxycinnamic acid (HCAD) concentrations in leaves of *Solanum tuberosum*, genotype VR808, under water stress and inoculation of arbuscular mycorrhizal fungi, in the second harvest. Where, WM: without mycorrhiza inoculation, CC: *Claroideoglomus claroideum*, HMC26: *Claroideoglomus lamellosum* and MIX: CC + HMC26; 0: normal irrigation; S1 and S2: levels of water stress; nd: no detected. Means followed by the same lowercase letter compare the difference in stress level within the same inoculation condition (Tukey 5%). Table S3: Individual flavonols (FLAV) and hydroxycinnamic acid (HCAD) concentrations in leaves of *Solanum tuberosum*, genotype CB2011-104, under water stress and inoculation of arbuscular mycorrhizal fungi, in the second harvest. Where, WM: without mycorrhiza inoculation, CC: *Claroideoglomus claroideum*, HMC26: *Claroideoglomus lamellosum* and MIX: CC + HMC26; 0: normal irrigation; S1 and S2: levels of water stress; nd: no detected. Means followed by the same lowercase letter compare the difference in stress level within the same inoculation condition (Tukey 5%). Table S4: Individual flavonols (FLAV) and hydroxycinnamic acid (HCAD) concentrations in leaves of *Solanum tuberosum*, genotype VR808, under water stress and inoculation of arbuscular mycorrhizal fungi, in the third harvest. Where, WM: without mycorrhiza inoculation, CC: *Claroideoglomus claroideum*, HMC26: *Claroideoglomus lamellosum* and MIX: CC + HMC26; 0: normal irrigation; S1 and S2: levels of water stress; nd: no detected. Means followed by the same lowercase letter compare the difference in stress level within the same inoculation condition (Tukey 5%). Table S5: Individual flavonols (FLAV) and hydroxycinnamic acid (HCAD) concentrations in leaves of *Solanum tuberosum*, genotype CB2011-104, under water stress and inoculation of arbuscular mycorrhizal fungi, in the third harvest. Where, WM: without mycorrhiza inoculation, CC: *Claroideoglomus claroideum*, HMC26: *Claroideoglomus lamellosum* and MIX: CC + HMC26; 0: normal irrigation; S1 and S2: levels of water stress; nd: no detected. Means followed by the same lowercase letter compare the difference in stress level within the same inoculation condition (Tukey 5%). Table S6: significance of *p*-values for the main effects and interaction for the variables measured and analyzed by means of factorial ANOVA to genotype VR808 in the first harvest. Table S7: significance of *p*-values for the main effects and interaction for the variables measured and analyzed by means of factorial ANOVA to genotype CB2011-104 in the first harvest. Table S8: significance of *p*-values for the main effects and interaction for the variables measured and analyzed by means of factorial ANOVA to genotype VR808 in the second harvest. Table S9: significance of *p*-values for the main effects and interaction for the variables measured and analyzed by means of factorial ANOVA to genotype CB2011-104 in the second harvest. Table S10: significance of *p*-values for the main effects and interaction for the variables measured and analyzed by means of factorial ANOVA to genotype VR808 in the third harvest. Table S11: significance of *p*-values for the main effects and interaction for the variables measured and analyzed by means of factorial ANOVA to genotype CB2011-104 in the third harvest. Figure S5. Lipid peroxidation in *Solanum tuberosum* leaves, under water stress and inoculation with arbuscular mycorrhizal fungi, genotype VR 808 s harvest (A), genotype VR 808 third harvest (B) genotype CB2011-104 s harvest (C), and genotype CB2011-104 third harvest (D). Means followed by the same lowercase letter compare the difference in stress level within the same inoculation condition (Tukey 5%). Where, WM: without mycorrhiza inoculation, CC: *Claroideoglomus claroideum*, HMC26: *Claroideoglomus lamellosum* and MIX: CC + HMC26; 0: normal irrigation; S1 and S2: levels of water stress. Table S12. Tuber numbers of *Solanum tuberosum*, genotype VR808, under water stress and inoculation of arbuscular mycorrhizal fungi. Means followed by the same lowercase letter compare the difference in stress level within the same inoculation condition (Tukey 5%). Where, WM: without mycorrhiza inoculation, CC: *Claroideoglomus claroideum*, HMC26: *Claroideoglomus lamellosum* and MIX: CC + HMC26; 0: normal irrigation; S1 and S2: levels of water stress. Table S13. Tuber numbers of *Solanum tuberosum*, genotype CB2011-104, under water stress and inoculation of arbuscular mycorrhizal fungi. Means followed by the same lowercase letter compare the difference in stress level within the same inoculation condition (Tukey 5%). Where, WM: without mycorrhiza inoculation, CC: *Claroideoglomus claroideum*, HMC26: *Claroideoglomus lamellosum* and MIX: CC + HMC26; 0: normal irrigation; S1 and S2: levels of water stress.
