# Supplementary material for: Physiological and Metabolic Effects of the Inoculation of Arbuscular Mycorrhizal Fungi in Solanum tuberosum Crops under Water Stress
Source: Plants (Basel). 2022 Sep 27;11(19):2539. doi: 10.3390/plants11192539 (PMC9570523; doi:10.3390/plants11192539)
Supplement: Supplementary file 1 [file plants-11-02539-s001.zip › plants-1903081-supplementary.pdf]

Supplementary material:

Table S1: Identification of phenolic compounds by HPLC-DAD-MS/MS in leaves of *Solanum tuberosum* under water stress and inoculation of arbuscular mycorrhizal fungi.

**Compound**	**t_R (min)**	**Tentative Identification**	**ʎ max (nm)**	**[M - H]-**	**Product ions**
HCAD1	5.9	5-caffeoylquinic acid	325	353.09	191.06; 161.02; 135.04
HCAD2	12.8	caffeoylquinic acid isomer	325	353.08	191.05; 175.11; 161.02; 133.0
HCAD3	16.4	caffeoylquinic acid isomer	325	353.08	191.05; 171.04; 135.04
HCAD4	19.5	caffeoylquinic acid isomer	325	353.08	191.06; 179.03; 135.04
HCAD5	20.6	no identified	290	-	-
HCAD6	24.5	no identified	320	-	-
FLAV1	13.4	no identified	350	-	-
FLAV2	14.5	quercetin-pentoside-rutinoside	351	741.18	300.1
FLAV3	15.4	no identified	351	-	-
FLAV4	16.1	quercetin-rutinoside	353	609.14	449.02; 301.12
FLAV5	17.9	kampferol-rutinoside	345	593.15	429.17; 285.02
FLAV6	21.6	no identified	351	429.17	-
FLAV7	24.1	no identified	352	431.19	333.10

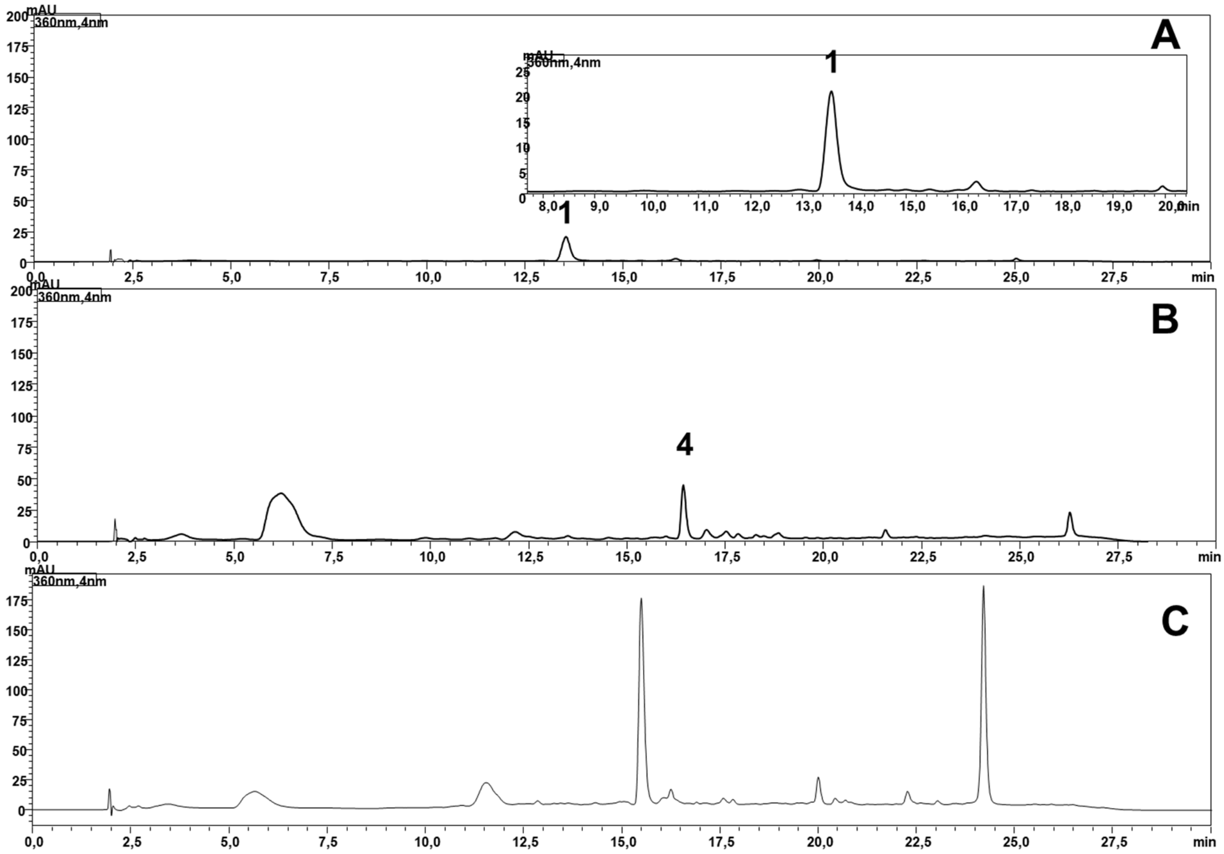

Figure S1: HPLC-DAD chromatogram (360 nm) for leaves of *Solanum tuberosum*, genotype VR808 under water stress and inoculation of arbuscular mycorrhizal fungi. Where: A: first harvest; B: second harvest; C: third harvest; 1: no identified; 4: quercetin-rutinoside (please Table S1)

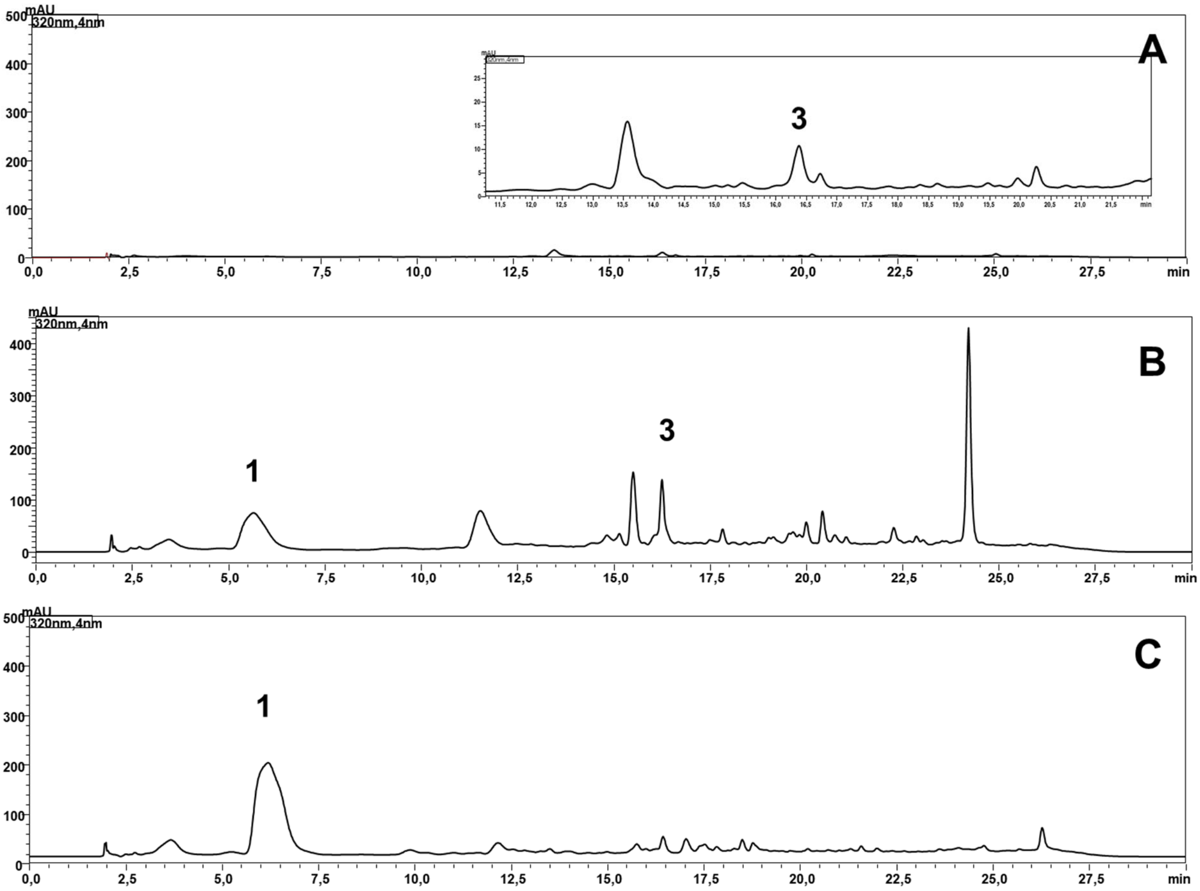

Figure S2: HPLC-DAD chromatogram (320 nm) for leaves of *Solanum tuberosum*, genotype VR808 under water stress and inoculation of arbuscular mycorrhizal fungi. Where: A: first harvest; B: second harvest; C: third harvest; 1: 5-caffeoylquinic acid 1; 3: caffeoylquinic acid isomer (please see Table S1)

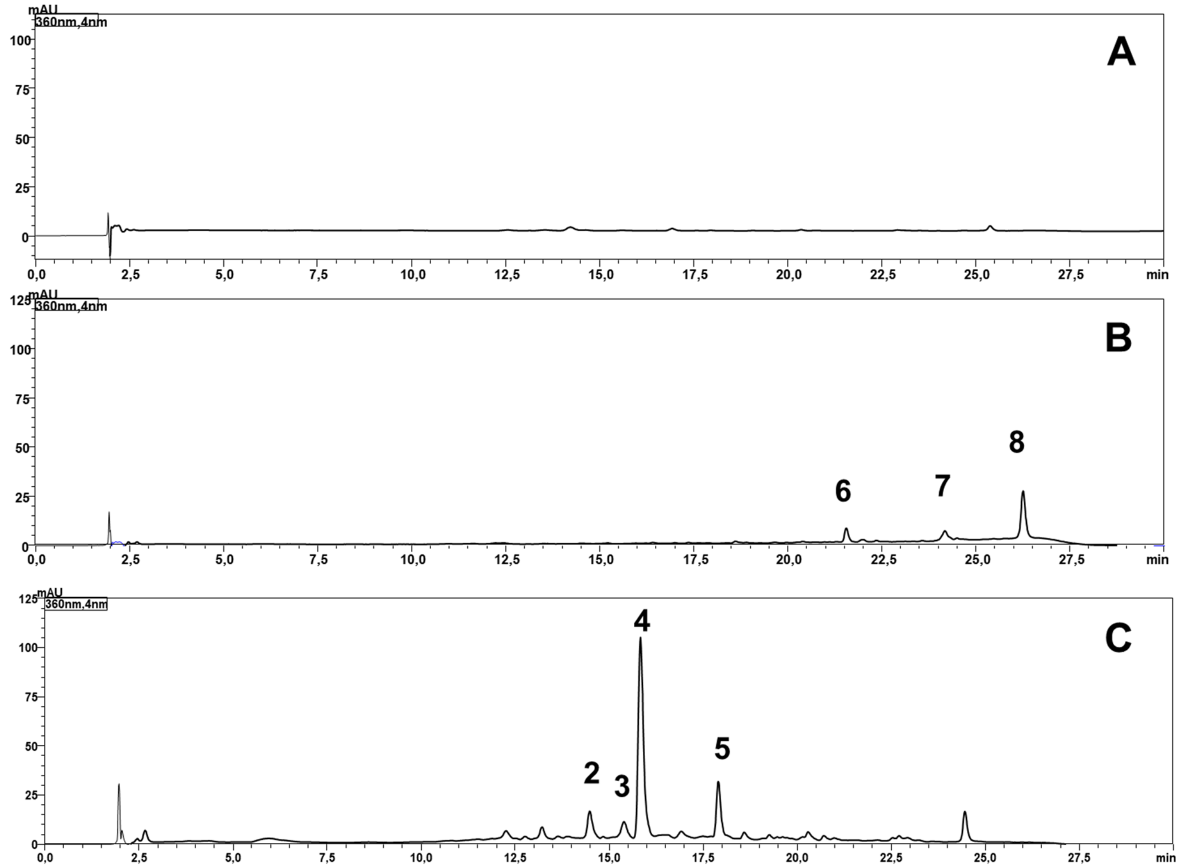

Figure S3: HPLC-DAD chromatogram (360 nm) for leaves of *Solanum tuberosum*, genotype CB2011-104 under water stress and inoculation of arbuscular mycorrhizal fungi. Where: A: first harvest; B: second harvest; C: third harvest; 2: quercetin-rutinoside-pentoside, 3: no identified, 4: quercetin-rutinoside, 5: kampferol-rutinoside, 6: no identified, 7: no identified (Please see Table S1)

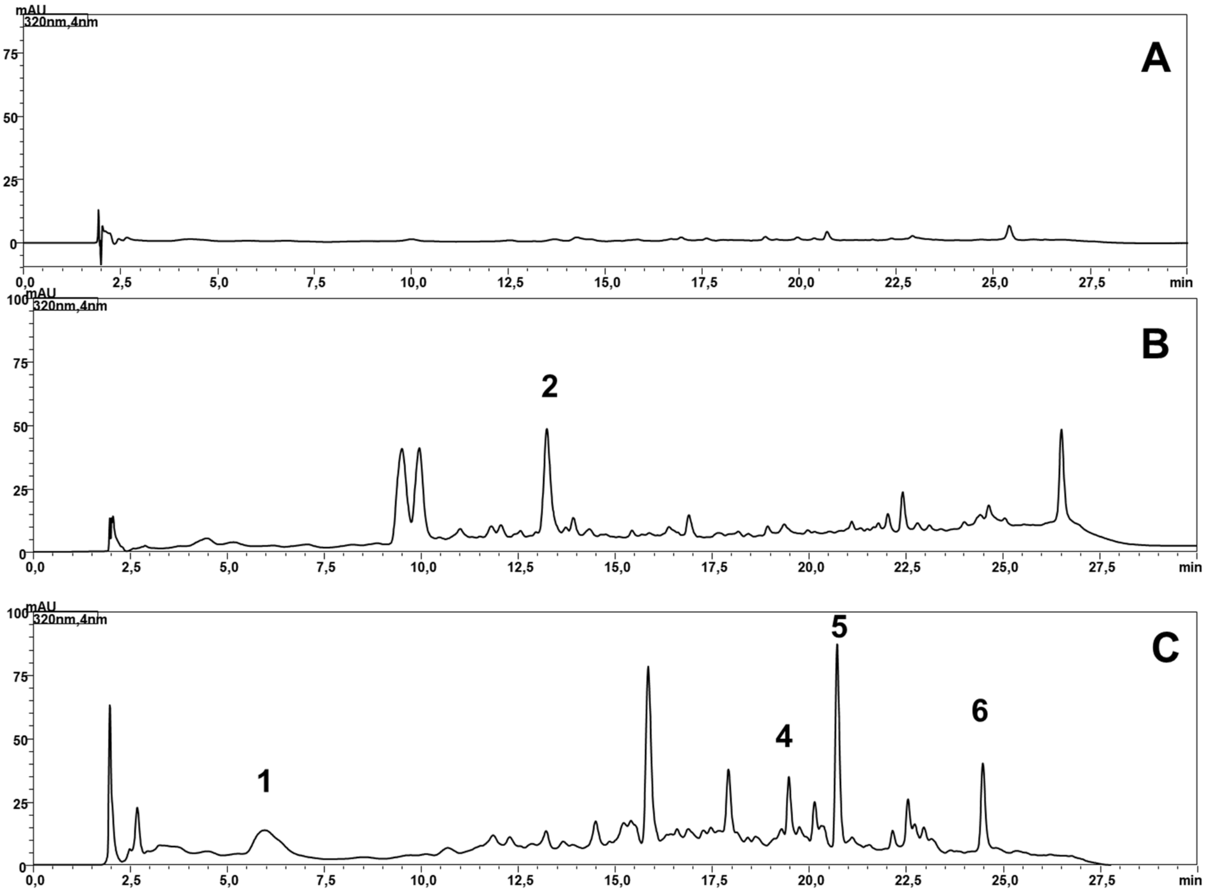

Figure S4: HPLC-DAD chromatogram (320 nm) for leaves of *Solanum tuberosum*, genotype CB2011-104 under water stress and inoculation of arbuscular mycorrhizal fungi. Where: A: first harvest; B: second harvest; C: third harvest; 1: 5-caffeoylquinic acid, 2: caffeoylquinic acid isomer, 3: caffeoylquinic acid isomer, 4: caffeoylquinic acid isomer, 5: no identified, 6: no identified (Please see Table S1)

Table S2: Individual flavonols (FLAV) and Hydroxycinnamic acid (HCAD) concentrations in leaves of *Solanum tuberosum*, genotype VR808 under water stress and inoculation of arbuscular mycorrhizal fungi, in the second harvest. Where, WM: without mycorrhiza inoculation, CC: *Claroideoglomus claroideum*, HMC26: *Claroideoglomus lamellosus* and MIX: CC + HMC26; 0: normal irrigation; S1 and S2: levels of water stress; nd: no detected. Means followed by the same lowercase letter compare the difference in stress level within the same inoculation condition (Tukey 5%). Identifications according to Table S1

AMF	Stress	FLAV4	HCAD1	HCAD3
WM	0	6.12 ± 1.64 *ab*	13.59±8.60 *c*	2.29±0.15 *b*
	S1	5.56 ± 2.81 a*bc*	59.73±56.21 *a*	0.18±0.01 *de*
	S2	3.43 ± 1.88 *cd*	3.44±1.54 *c*	0.96±0.02 *cd*
CC	0	2.42 ± 0.67 *d*	2.81±0.91 *c*	1.16±0.51 *c*
	S1	4.35 ± 1.84 *bcd*	58.96±72.20 *ab*	2.19±1.06 *b*
	S2	2.77 ± 1.33 *d*	1.79±0.47 *c*	nd
HMC26	0	7.50 ± 0.61 *a*	16.51±8.36 *bc*	nd
	S1	3.29 ± 1.19 *cd*	4.85±1.53 *c*	1.60±0.49 *bc*
	S2	2.46 ± 0.70 *d*	16.62±16.93 *bc*	*nd*
MIX	0	5.81 ± 2.61 *abc*	4.61±1.17 *c*	3.48±1.46 *a*
	S1	4.38 ± 1.52 *bcd*	4.54±1.56 *c*	nd
	S2	2.65 ± 0.19 *d*	3.82±1.67 *c*	1.29±0.29 *c*

Table S3: Individual flavonols (FLAV) and Hydroxycinnamic acid (HCAD) concentrations in leaves of *Solanum tuberosum*, genotype CB2011-104 under water stress and inoculation of arbuscular mycorrhizal fungi, in the second harvest. Where, WM: without mycorrhiza inoculation, CC: *Claroideoglomus claroideum*, HMC26: *Claroideoglomus lamellosus* and MIX: CC + HMC26; 0: normal irrigation; S1 and S2: levels of water stress; nd: no detected. Means followed by the same lowercase letter compare the difference in stress level within the same inoculation condition (Tukey 5%). Identifications according to Table S1

AMF	Stress	FLAV6	FLAV7	HCAD1	HCAD2
WM	0	nd	4.55±2.64 *ab*	5.00±1.80 a*bc*	2.89±0.93 *abc*
	S1	nd	6.78±3.03 *a*	5.87±4.64 *abc*	4.78±3.06 *ab*
	S2	1.12±0.09 *b*	3.77±0.78 *bc*	4.62±1.91 *abc*	3.84±0.49 *abc*
CC	0	nd	2.57±0.99 *bc*	10.99±11.39 *a*	4.95±4.61 *a*
	S1	nd	1.87±0.35 *c*	2.16±0.50 *bc*	1.57±0.52 *bc*
	S2	1.29±0.20 *a*	3.96±1.20 *bc*	nd	2.3±0.85 *abc*
HMC26	0	nd	2.14±0.99 *c*	2.71±1.10 *bc*	1.59±0.80 *bc*
	S1	nd	2.72±0.61 *bc*	3.27±2.50 *bc*	2.56±1.93 *abc*
	S2	1.12±0.10 *b*	3.20±1.12 *bc*	8.24±10.23 *ab*	4.17±3.21 *abc*
MIX	0	nd	3.10±1.31 *bc*	1.28±0.22 *bc*	1.40±0.38 *c*
	S1	nd	1.83±0.86 *c*	1.89±0.71 *bc*	1.37±0.54 *c*
	S2	nd	2.14±1.10 *c*	1.72±0.45 *bc*	2.66±1.68 *abc*

Table S4: Individual flavonols (FLAV) and Hydroxycinnamic acid (HCAD) concentrations in leaves of *Solanum tuberosum*, genotype VR808 under water stress and inoculation of arbuscular mycorrhizal fungi, in the third harvest. Where, WM: without mycorrhiza inoculation, CC: *Claroideoglomus claroideum*, HMC26: *Claroideoglomus lamellosus* and MIX: CC + HMC26; 0: normal irrigation; S1 and S2: levels of water stress; nd: no detected. Means followed by the same lowercase letter compare the difference in stress level within the same inoculation condition (Tukey 5%). Identifications according to Table S1.

AMF	Stress	FLAV4	HCAD1	HCAD2	HCAD3	HCAD4	HCAD6
WM	0	6.46±3.10 *ab*	20.73±19.98 *b*	5.13±3.31 *b*	6.29±3.95 *cde*	5.77±0.63 *abc*	32.70±3.17 *b*
	S1	3.06±1.50 *bcd*	8.84±6.24 *b*	nd	1.60±1.19 *f*	2.96±0.76 *de*	20.46±6.25 *b*
	S2	3.53±1.58 *bcd*	2.72±0.70 *b*	nd	7.52±2.94 *bcd*	5.02±1.71 *bcd*	23.87±9.80 *b*
CC	0	1.66±0.14 *d*	2.42±0.51 *b*	nd	3.80±2.10 *def*	4.08±3.10 *cde*	16.51±12.39 *b*
	S1	1.91±0.81 *cd*	5.03±0.77 *b*	nd	10.75±0.54 *ab*	8.07±0.43 *a*	32.41±6.52 *b*
	S2	3.13±1.46 *bcd*	4.02±1.31 *b*	nd	7.48±1.00 *bc*	6.35±0.90 *ab*	34.11±2.97 *ab*
HMC26	0	8.61±5.51 *ab*	47.55±48.88 *ab*	11.21±6.11 *a*	4.14±3.45 *cdef*	3.97±2.33 *cde*	29.31±16.92 *b*
	S1	5.79±3.21 *ab*	75.36±92.66 *a*	nd	12.34±4.01 *a*	6.94±1.48 *ab*	54.16±32.15 *a*
	S2	3.66±2.73 *bcd*	6.71±6.87 *b*	nd	5.21±3.69 *cdef*	2.32±1.28 *e*	17.51±15.88 *b*
MIX	0	3.74±1.15 *bcd*	3.06±1.04 *b*	nd	3.14±0.65 *ef*	3.89±0.60 *cde*	14.59±3.55 *b*
	S1	1.75±0.34 *d*	2.47±0.23 *b*	nd	2.85±1.34 *ef*	5.73±2.30 *abc*	19.08±9.51 *b*
	S2	5.65±2.16 *abc*	14.78±15.25 *b*	nd	7.98±2.12 *bc*	3.93±1.01 *cde*	29.73±10.29 *b*

Table S5: Individual flavonols (FLAV) and Hydroxycinnamic acid (HCAD) concentrations in leaves of *Solanum tuberosum*, genotype CB2011-104 under water stress and inoculation of arbuscular mycorrhizal fungi, in the third harvest. Where, WM: without mycorrhiza inoculation, CC: *Claroideoglomus claroideum*, HMC26: *Claroideoglomus lamellosus* and MIX: CC + HMC26; 0: normal irrigation; S1 and S2: levels of water stress; nd: no detected. Means followed by the same lowercase letter compare the difference in stress level within the same inoculation condition (Tukey 5%). Identifications according to Table S1.

AMF	Stress	FLAV2	FLAV3	FLAV4	FLAV5	HCAD1	HCAD4	HCAD5	HCAD6
WM	0	1.52±0.50 *a*	1.21±0.71 *a*	6.26±4.47 *ab*	2.40±1.02 *a*	29.80±26.16 *ab*	6.41±2.11 *bcd*	1.13±0.29 *cd*	12.17±6.70 *bc*
	S1	Nd	nd	nd	nd	5.80±2.99 *b*	4.22±0.89 *d*	2.83±0.58 *b*	9.82±0.49 *bc*
	S2	Nd	nd	nd	nd	2.41±1.99 *b*	5.20±1.55 *cd*	2.71±0.53 *bc*	10.95±6.06 *bc*
CC	0	Nd	nd	2.12±0.38 *bc*	nd	1.87±0.99 *b*	4.27±0.48 *d*	0.72±0.07 *d*	6.86±1.05 *c*
	S1	Nd	nd	nd	nd	1.27±0.11 *b*	6.97±1.85 *bcd*	3.83±1.37 *ab*	17.73±8.21 *abc*
	S2	Nd	nd	nd	1.36±0.23 *b*	18.90±8.37 *b*	9.96±7.69 *ab*	4.69±2.65 *a*	26.92±22.09 *a*
HMC26	0	Nd	nd	1.88±0.71 *bc*	nd	12.48±14.54 *b*	3.75±1.44 *d*	0.70±0.11 *d*	6.43±2.13 *c*
	S1	Nd	nd	nd	nd	23.18±24.27 *ab*	6.76±0.91 *bcd*	3.88±0.85 *ab*	19.97±7.26 *ab*
	S2	Nd	nd	4.19±3.66 *abc*	1.15±0.06 *b*	1.48±0.31 *b*	12.88±2.24 *ab*	4.81±1.25 *a*	27.40±7.14 *a*
MIX	0	Nd	nd	7.66±9.24 *a*	1.74±0.93 *ab*	75.76±110.3 *a*	6.70±1.52 *bcd*	1.13±0.13 *cd*	13.18±3.55 *bc*
	S1	Nd	nd	nd	nd	1.53±0.50 *b*	3.60±1.06 *d*	2.34±0.44 *bcd*	5.28±0.24 *c*
	S2	Nd	nd	2.59±1.13 *bc*	1.32±0.34 *b*	3.33±1.89 *b*	8.71±2.86 *abc*	5.34±1.02 *a*	14.70±6.92 *a*

Table S6: significance of *p*-values for the main effects and interaction for the variables measured and analyzed by means of factorial ANOVA to genotype VR808 in the first harvest.

**Variable**	**Mycorrhiza**	**Stress**	**Interactions (M x S)**
FLAV1	***	***	***
HCAD3	***	***	***
TOTALPHENOLICS	ns	***	***
DPPH	ns	***	ns
TEAC	***	ns	***
CUPRAC	***	***	***
FRAP	***	***	ns

ns, not significant
***$p < 0.01$

Table S7: significance of *p*-values for the main effects and interaction for the variables measured and analyzed by means of factorial ANOVA to genotype CB2011-104 in the first harvest.

**Variable**	**Mycorrhiza**	**Stress**	**Interactions (M x S)**
TOTALPHENOLICS	***	***	***
DPPH	***	***	***
TEAC	ns	***	ns
CUPRAC	***	***	***
FRAP	***	***	***

ns, not significant
***p < 0.01

Table S8: significance of *p*-values for the main effects and interaction for the variables measured and analyzed by means of factorial ANOVA to genotype VR808 in the second harvest.

**Variable**	**Mycorrhiza**	**Stress**	**Interactions (M x S)**
FLAV4	***	***	***
FLAVTOT	***	***	***
HCAD1	***	***	***
HCAD3	***	***	***
HCADTOT	***	***	***
TOTALPHENOLICS	***	***	***
DPPH	***	***	***
TEAC	ns	***	***
CUPRAC	ns	***	***
FRAP	***	***	***

ns, not significant
***$p < 0.01$

Table S9: significance of *p*-values for the main effects and interaction for the variables measured and analyzed by means of factorial ANOVA to genotype CB2011-104 in the second harvest.

Variable	Mycorrhiza	Stress	Interactions (M x S)
FLAV6	***	***	***
FLAV7	***	ns	***
FLAVTOT	***	***	***
HCAD1	***	ns	***
HCAD2	***	ns	***
HCADTOT	***	ns	***
TOTALPHENOLICS	***	***	ns
DPPH	***	***	***
TEAC	***	***	***
CUPRAC	***	***	***
FRAP	***	***	***

ns, not significant
***$p < 0.01$

Table S10: significance of *p*-values for the main effects and interaction for the variables measured and analyzed by means of factorial ANOVA to genotype VR808 in the third harvest.

Variable	Mycorrhiza	Stress	Interactions (M x S)
FLAV4	***	***	***
FLAVTOT	***	***	***
HCAD1	***	ns	***
HCAD2	***	***	***
HCAD3	***	***	***
HCAD4	***	***	***
HCAD6	***	***	***
HCADTOT	***	ns	***
TOTALPHEN	ns	***	***
DPPH	ns	***	***
TEAC	ns	***	***
CUPRAC	***	***	***
FRAP	ns	***	***

ns, not significant
***$p < 0.01$

Table S11: significance of *p*-values for the main effects and interaction for the variables measured and analyzed by means of factorial ANOVA to genotype CB2011-104 in the third harvest.

Variable	Mycorrhiza	Stress	Interactions (M x S)
FLAV2	***	***	***
FLAV3	***	***	***
FLAV4	***	***	***
FLAV5	***	***	***
FLAVTOT	***	***	***
HCAD1	***	***	***
HCAD4	***	***	***
HCAD5	***	***	***
HCAD6	***	***	***
HCADTOT	ns	ns	***
TOTALPHEN	***	***	***
DPPH	ns	ns	***
TEAC	***	***	***
CUPRAC	***	***	***
FRAP	ns	***	***

ns, not significant
***$p < 0.01$

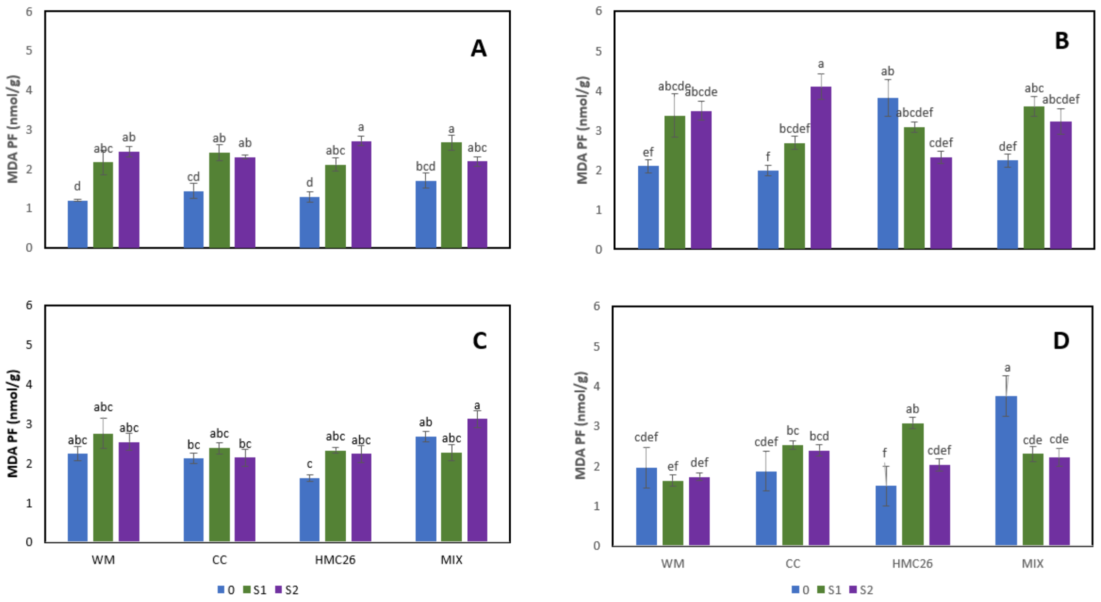

Figure S5. Lipid peroxidation in *Solanum tuberosum* leaves, under water stress and inoculation with arbuscular mycorrhizal fungi, genotype VR 808 second harvest (A), genotype VR 808 third harvest (B) genotype CB2011-104 second harvest (C), and genotype CB2011-104 third harvest (D). Means followed by the same lowercase letter compare the difference in stress level within the same inoculation condition (Tukey 5%). Where, WM: without mycorrhiza inoculation, CC: *Claroideoglomus claroideum*, HMC26: *Claroideoglomus lamellosum* and MIX: CC + HMC26; 0: normal irrigation; S1 and S2: levels of water stress.

Table S12. Tuber numbers of *Solanum tuberosum*, genotype VR808 under water stress and inoculation of arbuscular mycorrhizal fungi. Means followed by the same lowercase letter compare the difference in stress level within the same inoculation condition (Tukey 5%). Where, WM: without mycorrhiza inoculation, CC: *Claroideoglomus claroideum*, HMC26: *Claroideoglomus lamellosum* and MIX: CC + HMC26; 0: normal irrigation; S1 and S2: levels of water stress

	**0**	**S1**	**S2**
WM	7 ± 1 *a*	8 ± 1 *a*	5 ± 0 *bc*
CC	9 ± 1 *a*	9 ± 0 *a*	7 ± 0 *ab*
HMC26	7 ± 1 *ab*	8 ± 0 *a*	4 ± 0 *c*
MIX	8 ± 1 *a*	5 ± 0 *bc*	5 ± 0 *c*

Table S13. Tuber numbers of *Solanum tuberosum*, genotype CB2011-104 under water stress and inoculation of arbuscular mycorrhizal fungi. Means followed by the same lowercase letter compare the difference in stress level within the same inoculation condition (Tukey 5%). Where, WM: without mycorrhiza inoculation, CC: *Claroideoglomus claroideum*, HMC26: *Claroideoglomus lamellosum* and MIX: CC + HMC26; 0: normal irrigation; S1 and S2: levels of water stress

	**0**	**S1**	**S2**
WM	16 ± 3 *ab*	7 ± 1 *ab*	7 ± 5 *b*
CC	20 ± 4 *ab*	15 ± 5 *ab*	11 ± 1 *ab*
HMC26	14 ± 4 *ab*	22 ± 4 *ab*	12 ± 3 *ab*
MIX	25 ± 5 *a*	14 ± 4 *ab*	12 ± 4 *ab*
